# Time of Clinic Appointment and Serious Illness Communication in Oncology

**DOI:** 10.1177/10732748231170488

**Published:** 2023-04-18

**Authors:** Likhitha Kolla, Jinbo Chen, Ravi B. Parikh

**Affiliations:** 1 Perelman School of Medicine, Department of Biostatistics, Epidemiology and Informatics, 14640 University of Pennsylvania, Philadelphia, PA, USA; 2 Perelman School of Medicine, Department of Medicine, 14640 University of Pennsylvania, Philadelphia, PA, USA; 3 Perelman School of Medicine, Department of Medical Ethics and Health Policy, 14640 University of Pennsylvania, Philadelphia, PA, USA; 4 Corporal Michael J. Crescenz VA Medical Center, Philadelphia, PA, USA

**Keywords:** Palliative care, quality of life, oncology, health care, decision making

## Abstract

**Introduction:**

Serious illness communication in oncology increases goal concordant care. Factors associated with the frequency of serious illness conversations are not well understood. Given prior evidence of the association between suboptimal decision-making and clinic time, we aimed to investigate the relationship between appointment time and the likelihood of serious illness conversations in oncology.

**Methods:**

We conducted a retrospective study of electronic health record data from 55 367 patient encounters between June 2019 to April 2020, using generalized estimating equations to model the likelihood of a serious illness conversation across clinic time.

**Results:**

Documentation rate decreased from 2.1 to 1.5% in the morning clinic session (8am-12pm) and from 1.2% to .9% in the afternoon clinic session (1pm-4pm). Adjusted odds ratios for Serious illness conversations documentation rates were significantly lower for all hours of each session after the earliest hour (adjusted odds ratios .91 [95% CI, .84-.97], *P* = .006 for overall linear trend).

**Conclusions:**

Serious illness conversations between oncologists and patients decrease considerably through the clinic day, and proactive strategies to avoid missed conversations should be investigated.

## Introduction

Early serious illness communication in oncology increases goal-concordant care, decreases clinician and caregiver moral distress, and increases hospice near the end of life.^1,2^ Serious illness conversations (SIC) are discussions between clinicians and patients about the progression of an advanced health condition that could adversely strain the patient’s quality of life or their caregivers’. These conversations typically inquire the patient’s knowledge of their illness and prognosis, deliver new prognostic information, and explore next steps of medical care consistent with the patient’s goals and priorities.^1^ Organizations including the National Coalition for Hospice and Palliative Care and the National Comprehensive Cancer Network recommend that oncologists initiate serious illness discussions for patients early in the cancer care continuum.^3^ In addition, a majority of surveyed patients and caregivers in oncology prefer earlier and more in-depth goals of care conversations.^4^ However, serious illness communication generally does not occur until approximately 1 month before death – if at all.^3^

Time pressures and decision fatigue during a busy clinic day may be contributing reasons that prevent clinicians from engaging in necessary conversations.^5,6^ Aspects of care that are not immediately urgent may be omitted when clinicians fall behind in their schedule throughout the day. Furthermore, as the day progresses, physicians experience a progressive inability to continue making difficult decisions after having made many already, referred to as decision fatigue.^6^ Decision fatigue is well characterized in primary care settings. For example, studies have reported that time of day is associated with lower rates of flu vaccinations, cancer screening referrals, and statin prescriptions, as well as greater unwarranted antibiotic and opioid orders by primary care physicians.^7-11^ However, decision fatigue in serious illness communication has not been described.

Given prior evidence of suboptimal clinician decision-making in non-oncology settings in latter parts of a clinic day or session, we investigated the association between appointment time and likelihood of serious illness conversations. Our work expands knowledge on the frequency and nature of serious illness conversations; and informs interventions that promote proactive communication and goal-concordant cancer care delivery.

## Methods

This work presents a secondary post-hoc analysis of a randomized clinical trial (NLM, NCT03984773) among 75 clinicians and 17 696 patients with cancer.^12^ Results from the trial were previously published.^13^ The trial was conducted between July 2019 to April 2020 and investigated the use of machine-generated mortality predictions and behavioral nudges to clinicians to promote serious illness conversations. The trial protocol and overall study was granted approval by The University of Pennsylvania Institutional Review Board with a waiver of written informed consent. We merged billing and institutional electronic health record (EHR) data from Clarity, an EPIC® reporting database, to identify a cohort of medical oncology encounters from 1 of 9 medical oncology clinics (8 disease-specific clinics within a tertiary practice, 1 general oncology clinic) within a large academic healthcare system. We studied return patient encounters with a clinician (medical oncology physician, nurse practitioner, or physician assistant) from June 17, 2019 to April 17, 2020. New patient encounters, encounters with clinicians with <40 total appointments during the study period, and encounters after the first documented SIC within the study period (Supplementary Figure S1).

While SIC conversations were not audio recorded, SIC documentation was used as a surrogate for serious illness communication, as SIC documentation is a quality metric used by many organizations including the American Society for Clinical Oncology’s Quality Oncology Practice Initiative and the Centers for Medicare and Medicaid Services Oncology Care Model; and has been used to define SIC conversations in prior work.^2,14^ We ascertained the presence of a SIC from either (1) a specific SIC note type in the EHR, or (2) an SIC smart phrase in clinical progress notes. A smart phrase is a pre-built template or shortcut for entering commonly used phrases, sentences, or paragraphs into a patient’s EHR record. Smart phrases are designed to save time and increase efficiency in documenting patient encounters. The ACP smart phrase in the EHR is customized to pull a pre-defined SIC template, created by the Ariadne Labs, into the note.^14^

Appointment times between 8am and 4pm were separated by the hour. For example, all appointments between 8am and 8:59am were assigned to 8am. Visits before 8am and after 4pm were grouped with the 8am and 4pm timepoints, respectively. Oncology clinicians (ie medical oncology physician, nurse practitioner, or physician assistant) in eligible clinics practiced in either a morning (8am to 11am) or afternoon (12pm to 4pm) session and could alternate between morning and afternoon sessions on different days. Time was indicated by grouping appointment times in the order they occur in a session (eg 8am and 12pm were grouped as hour 1). Advanced practice providers (APPs), including physician assistants and nurse practitioners, were consistently assigned to oncology physicians, with a ratio of oncologists to APPs ranging from 1:1 to 2:1.

We use the generalized estimating equation approach, clustering by individual clinician, to estimate the probability of SIC documentation.^15^ Session hour (1-5) was included as a categorical variable to calculate the relative odds of documentation for each hour of a session after the first and as a continuous variable for assessing overall linear time trend. We adjusted for patient age, race, ethnicity, and gender, insurance, tumor type and stage, Charlson comorbidity count, and appointment month/year. As the study period coincided with a quality improvement effort to prompt conversations among patients at risk of short-term mortality, we additionally adjusted for whether a patient’s clinician received a conversation prompt for a specific encounter.^13^ To evaluate potential bias from the morning to afternoon session transition, we performed a sensitivity analysis using a restricted sample that excluded encounters from 12pm. Our final model includes 11 independent variables and is trained on 55367 observations, which exceeds the minimum recommended sample size for an observational multivariable regression analysis of at least 650 (n = 100 + 50*i, i* = 11).^16^ Two-sided Wald tests were used to test all hypotheses with *P* < .05 indicating statistical significance. Analyses were performed between December 2021 and February 2023 using R, version 4.0.3. The reporting of the study conforms to STROBE guidelines.^17^

## Results

**Figure 1.**
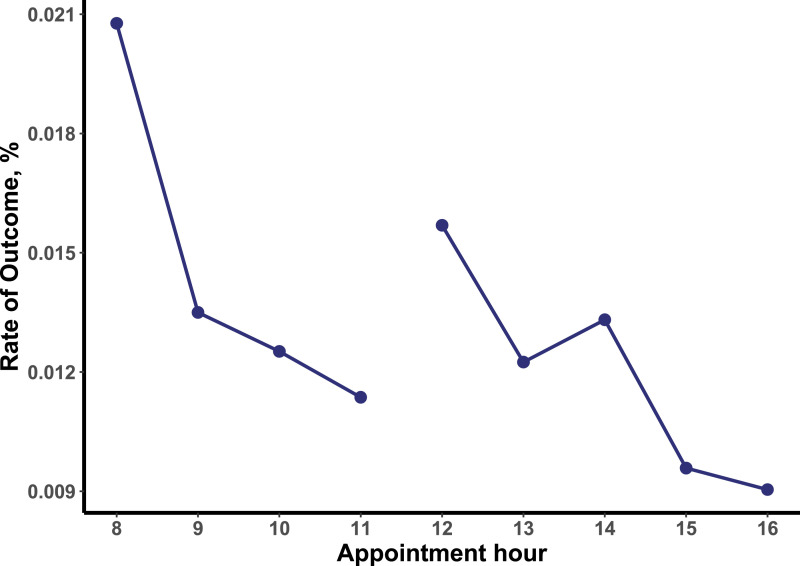
SIC rates by appointment time. Legend: Absolute serious illness communication rates by oncology clinic appointment hour. 8 – 11 represents typical morning session; 12 – 16 represents typical afternoon session.

The sample consisted of 75 oncology clinicians and 55 367 encounters with 17 696 patients. Patients’ mean (SD) age was 61.9 (14.0) years; 53.6% were female and 70.6% were non-Hispanic White. Compared to patients without SICs, patients with SICs were more likely to have gastrointestinal (14.1% vs 26.8%) and thoracic (12.6% vs 24.7%) malignancies and to carry Medicare insurance (47.0% vs 57.4%); other demographic characteristics were similar (Table 1). Unadjusted SIC documentation rates in the morning and afternoon sessions are displayed in Figure 1. Documentation rate decreased from 2.1 to 1.5% in the morning clinic session (8am-12pm) and from 1.2% to .9% in the afternoon clinic session (1pm-4pm). Adjusted odds ratios (ORs) for SIC documentation rates were significantly lower for all hours of each session after the earliest hour (adjusted OR .91 [95% CI, .84-.97], *P* = .006 for overall linear trend) (Table 2, Table 3). Results were consistent in the sensitivity analysis (adjusted OR .90 [95% CI, .83-.97], *P* = .007 for overall linear trend).

**Table 1.** Descriptive statistics of patient population.

	SIC Documentation		Overall (N = 55 367)
	No (N = 54 635)	Yes (N = 732)	
Age (years)			
Mean (SD)	61.8 (14.0)	66.0 (12.4)	61.9 (14.0)
Median [Min, Max]	63.6 [16.1, 101]	67.0 [19.1, 96.1]	63.7 [16.1, 101]
Sex			
Female	29 373 (53.8%)	364 (49.7%)	29 737 (53.7%)
Male	25 262 (46.2%)	368 (50.3%)	25 630 (46.3%)
Race			
Asian	1 748 (3.2%)	27 (3.7%)	1 775 (3.2%)
Non-Hispanic Black	11 061 (20.2%)	152 (20.8%)	11 213 (20.3%)
Hispanic	1 150 (2.1%)	16 (2.2%)	1 166 (2.1%)
Unknown	2 097 (3.8%)	23 (3.1%)	2 120 (3.8%)
Non-Hispanic White	38 579 (70.6%)	514 (70.2%)	39 093 (70.6%)
Tumor type			
Breast	7 852 (14.4%)	38 (5.2%)	7 890 (14.3%)
Central Nervous system + Melanoma	3 908 (7.2%)	64 (8.7%)	3 972 (7.2%)
Gastrointestinal	7 696 (14.1%)	196 (26.8%)	7 892 (14.3%)
General oncology	11 765 (21.5%)	133 (18.2%)	11 898 (21.5%)
Genitourinary	44 97 (8.2%)	31 (4.2%)	4 528 (8.2%)
Lymphoma	54 37 (10.0%)	36 (4.9%)	5 473 (9.9%)
Myeloma	6 608 (12.1%)	53 (7.2%)	6 661 (12.0%)
Thoracic	6 872 (12.6%)	181 (24.7%)	7 053 (12.7%)
Insurance			
Commercial	25 155 (46.0%)	263 (35.9%)	25 418 (45.9%)
Medicaid	3 812 (7.0%)	49 (6.7%)	3 861 (7.0%)
Medicare	25 668 (47.0%)	420 (57.4%)	26 088 (47.1%)
CCI			
Mean (SD)	4.54 (3.18)	6.58 (3.21)	4.57 (3.19)
Median [Min, Max]	3.00 [0, 18.0]	8.00 [0, 16.0]	3.00 [0, 18.0]
Conversation prompt			
False	49 730 (91.0%)	479 (65.4%)	50 209 (90.7%)
True	4 883 (8.9%)	230 (31.4%)	5 113 (9.2%)
Unknown	22 (.0%)	23 (3.1%)	45 (.1%)
Tumor stage			
Stage IV	3 621 (6.6%)	107 (14.6%)	3 728 (6.7%)
Other/Unknown	51 014 (93.4%)	625 (85.4%)	51 639 (93.3%)

**Table 2.** Adjusted odds of serious illness communication by session hour.

^a^Main Model			^b^Sensitivity Analysis (excluding 12pm)		
Hour	Adjusted OR (95% CI)	*P* Value	Hours	Adjusted OR	*P* Value
1	1.00 (Reference)		1	1.00 (Reference)	
2	.79 (.65-.93)	.03	2	.87 (.70-1.07)	.21
3	.80 (.66-.95)	.05	3	.80 (.64-1.00)	.05
4	.67 (.58-.86)	.001	4	.72 (.57-.92)	.01
5	.86 (.85-.96)	.60			
Overall time trend	.91 (.84-.97)	.006	Overall time trend	.90 (.83-.97)	.007

Note: OR = Odds ratio. Hour represents hour within a morning (8-11am) or afternoon (12-4pm) session.

^a^Models were adjusted for patient age, race, ethnicity, gender, insurance, tumor type and stage, Charlson comorbidity count, appointment month and year, and whether a conversation prompt was given for the encounter.

^b^Model does not include 12pm.

**Table 3.** Multivariable logistic regression for serious illness conversation outcome.

	Coefficient estimate	Odds ratio (95% CI)	*P*-Value
Appointment hour			
2	−.23	.79 (.63-.97)	.03
3	−.21	.8 (.64-.99)	.05
4	−.39	.67 (.53-.84)	.001
5	−.14	.86 (.49-1.48)	0.6
Race			
Black	−.08	.92 (.59-1.39)	0.7
Hispanic	.07	1.07 (.55-2.01)	.82
Unknown	−.18	.84 (.47-1.48)	.53
White	−0.2	.82 (.54-1.21)	.32
Age	.02	1.02 (1.01-1.03)	<.001
Sex	−.05	.95 (.80-1.12)	.55
Insurance			
Medicaid	.17	1.19 (.84-1.65)	.31
Medicare	1 (.81-1.23)	.96
CCI	.12	1.13 (1.10-1.16)	<.001
Conversation prompt (True)	1.27	3.56 (2.96-4.23)	<.001
Stage IV	.42	1.52 (1.23-1.96)	<.001
Tumor Body Site			
CNS + Melanoma	1.29	3.63 (2.30-5.40)	<.001
Gastrointestinal	1.44	4.22 (2.84-5.89)	<.001
General Oncology	1.02	2.77 (1.94-4.03)	<.001
Genitourinary	0.2	1.22 (.74-2.04)	.44
Lymphoma	.68	1.97 (1.26-3.19)	.004
Myeloma	.61	1.84 (1.20-2.86)	.006
Thoracic	1.42	4.14 (2.73-5.67)	<.001
Year	−.14	.87 (.57-1.29)	.49
Month	−.01	.99 (.95-1.03)	.76

## Discussion

Oncology clinicians’ likelihood of having and documenting serious illness conversations decreases as a clinic session progresses. Falling behind schedule and decision fatigue could be contributing reasons for this effect.^5-11^ Serious illness conversations involve coordinated efforts between the patients, their healthcare team, and their families to discuss disease trajectory and goals of care. These conversations can require significant time commitments and can be hard to prioritize if delays interfere with clinician schedules. Furthermore, SICs in oncology often cover emotionally charged topics, like unfavorable treatment outcomes, and can discourage clinicians from engaging in such hard conversations towards the end of a clinic session as they experience decision fatigue. Ultimately, lower rates of discussions about goals of care later in a session could result in more aggressive treatment regimens and ICU admissions near end-of-life, as well as fewer hospice referrals.^2^

This work expands the literature on how time of day affects clinician decision-making; and mechanisms for delayed serious illness conversations in oncology which could reason the paucity of similar discussions in adjacent fields.^18^ We acknowledge some methodological limitations that should be considered in the interpretation of our results. While we only examined return visits and adjusted for important metrics of patient severity, we could not account for unmeasured confounders such as patient and family wishes in this observational study. However, by clustering at the level of the oncologist, we accounted for clinician-specific variation in scheduling and patient risk. Furthermore, the cohort in this observational study constitutes clinicians and patients from a single academic institution where the clinicians had been trained a priori on specific SIC EHR documentation practices, which may restrict the generalizability of our results. We also acknowledge that physicians and patients may have had serious illness conversations without documentation or were not documented using the serious illness conversation template in the EHR, and the frequency of these conversations may be underreported. Finally, a low baseline rate of SICs could limit the conclusions drawn from the study. Although, our findings were similar to rates measured in prior studies (1.9% - 4.9%).^13^ The low rate could be explained by the inclusion of all patients, not just decedents, in our analyses; and by the nature of SICs, which are more in-depth and can take longer than traditional advance care planning conversations.

## Conclusion

Efforts to improve the quality of care should recognize the time pressure on patients and physicians, the effects of behavioral interventions, and the time costs of improving patient-physician communication. Several straightforward practice changes could address these time pressures. Proactive scheduling of high-risk patients earlier in a clinic session or scheduling separate visits for serious illness communication could facilitate necessary conversations and should be further studied. Alternatively, democratizing serious illness communication to other members of the health care team – including lay health workers – may offload clinicians who are under time pressures from potentially low-quality or missed serious illness communication.^19^ Future work should study the downstream effects of time-based decisions for serious illness conversations on end-of-life outcomes (ie chemotherapy treatments in the last 14 days of life, ICU admissions in the last 30 days, and late or non-referrals to hospice). In conclusion, oncologist-patient serious illness communication decreases considerably through the clinic day, reflecting potential time pressures and decision fatigue that warrant proactive strategies to avoid missed conversations.

## Supplemental Material

Supplemental material - Time of Clinic Appointment and Serious Illness Communication in Oncology Click here for additional data file. Supplemental material for Supplemental material - Time of Clinic Appointment and Serious Illness Communication in Oncology by Likhitha Kolla, Jinbo Chen and Ravi B. Parikh in Cancer Control Journal
