# Supplementary material for: Time of Clinic Appointment and Serious Illness Communication in Oncology
Source: Cancer Control. 2023 Apr 18;30:10732748231170488. doi: 10.1177/10732748231170488 (PMC10126780; doi:10.1177/10732748231170488)
Supplement: Supplemental material - Time of Clinic Appointment and Serious Illness Communication in Oncology [file sj-pdf-1-ccx-10.1177_10732748231170488.pdf]

Supplementary Information

Supplementary Figure 1: Flow diagram of patient encounter inclusion/exclusion criteria.

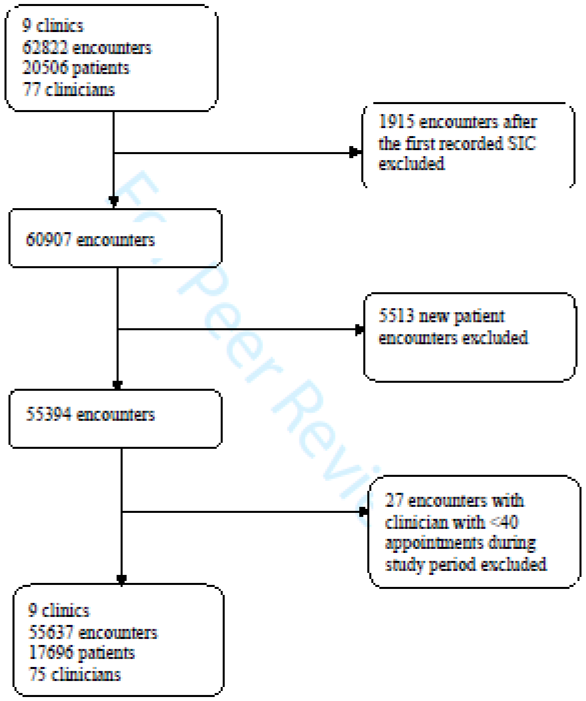

Supplementary Table 1: Adjusted odds ratios (OR) of serious illness communication by session hour from GEE models additionally adjusting for number of visits to date

[a]Main Model			[b]Sensitivity Analysis (excluding 12pm)		
Hour	Adjusted OR (95% CI)	*P* Value	Hours	Adjusted OR	*P* Value
1	1.00 (Reference)		1	1.00 (Reference)	
2	.79 (.64-.98)	.03	2	.87 (.71-1.07)	.21
3	.82 (.65-1.00)	.05	3	.81 (.65-1.00)	.05
4	.68 (.54-.86)	.001	4	.73 (.57-.93)	.01
5	.87 (.50-1.52)	.63			

Overall time trend	.91 (.84-.97)	.007	Overall time trend	.90 (.84-.97)	.008

NOTE: OR = Odds ratio. Hour represents hour within a morning (8-11 AM) or afternoon (12-4 PM) session.

[a]Models were adjusted for patient age, race, ethnicity, gender, insurance, tumor type and stage, Charlson comorbidity count, appointment month and year, whether a conversation prompt was given for the encounter, and number of visits to date.

[b]Model does not include 12pm.

Supplementary Table 2: Adjusted odds ratios (OR) of serious illness communication by session hour from GEE models trained on data with encounters after first SIC

[a]Main Model			[b]Sensitivity Analysis (excluding 12pm)		
Hour	Adjusted OR (95% CI)	*P* Value	Hours	Adjusted OR	*P* Value
1	1.00 (Reference)		1	1.00 (Reference)	
2	.70 (.58-.84)	<.001	2	.83 (.68-.99)	.04
3	.76 (.63-.91)	.003	3	.80 (.66-.96)	.02
4	.68 (.56-.83)	<.001	4	.78 (.64-.96)	.02
5	.78 (.48-1.29)	.35			
Overall time trend	.91 (.86-.97)	.003	Overall time trend	.92 (.86-.98)	.01

Note: OR, Odds ratio. Hour represents hour within a morning (8-11am) or afternoon (12-4pm) session.

[a]Models were adjusted for patient age, race, ethnicity, gender, insurance, tumor type and stage, Charlson comorbidity count, appointment month and year, and whether a conversation prompt was given for the encounter.

[b]Model does not include 12pm.
